# Modeling and Simulation of Mechanical Performance in Textile Structural Concrete Composites Reinforced with Basalt Fibers

**DOI:** 10.3390/polym14194108

**Published:** 2022-09-30

**Authors:** Rajesh Kumar Mishra, Bijoya Kumar Behera, Vijay Chandan, Shabnam Nazari, Miroslav Muller

**Affiliations:** 1 Department of Material Science and Manufacturing Technology, Faculty of Engineering, Czech University of Life Sciences Prague, 16500 Prague, Czech Republic; 2 Department of Textile Engineering, Indian Institute of Technology, New Delhi 110016, India

**Keywords:** fiber-reinforced concrete, basalt, finite element analysis (FEA), stress, strain energy, ductility

## Abstract

This investigation deals with the prediction of mechanical behavior in basalt-fiber-reinforced concrete using the finite element method (FEM). The use of fibers as reinforcement in concrete is a relatively new concept which results in several advantages over steel-reinforced concrete with respect to mechanical performance. Glass and polypropylene (PP) fibers have been extensively used for reinforcing concrete for decades, but basalt fibers have gained popularity in recent years due to their superior mechanical properties and compatibility with concrete. In this study, the mechanical properties of basalt-fiber-reinforced concrete are predicted using FEM analysis, and the model results are validated by conducting experiments. The effect of fiber-volume fraction on the selected mechanical performance of concrete is evaluated in detail. Significant improvement is observed when the loading is increased. There are superior mechanical properties, e.g., load bearing and strain energy in basalt-fiber-reinforced concrete as compared to conventional concrete slabs reinforced with gravel or stones. The results of the simulations are correlated with experimental samples and show a very high similarity. Basalt-fiber-reinforced concrete (BFRC) offers a lightweight construction material as compared to steel-fiber-reinforced concrete (SFRC). Further, the problem of corrosion is overcome by using this novel fiber material in concrete composites.

## 1. Introduction

Fiber-reinforced composites have been utilized for quite some time now. Composites using fibrous reinforcement materials with high strength, low density, along with high ductility are used in many fields such as aerospace, construction, and defense. Due to their evident advantages, much research has been carried out in this field to examine the possibility of using such composites in other fields where they have not been used. This approach also helps in decreasing the cost of materials for more widespread applications. Fiber-reinforced concrete, as opposed to conventional concrete, is a relatively new concept. The use of fibers in concrete decreases the weight of the slabs and columns. They also have very good insulation properties and are not magnetic in nature. Though at lower loads the crack widths and extensions are higher for fiber-reinforced concrete due to its lower Young’s modulus, at higher loads, crack spacing decreases relative to steel reinforcement. Creep and shrinkage behavior in fiber-reinforced concrete is similar to those in steel-reinforced concrete slabs and columns [1,2]. The fibers used for this purpose are glass, polypropylene (PP), basalt, and carbon fibers (though rarely, due to their relatively higher cost) [3,4,5,6,7].

Basaltic rock is formed from solidified lava (igneous rock). It is an extremely hard, fine-grained extrusive rock and consists of the minerals SiO_2_, Al_2_O_3_, CaO, MgO, K_2_O, Na_2_O, Fe_2_O_3_ and FeO. It has high abrasion resistance, excellent resistance to extreme temperatures, good insulation properties (heat, sound, and electrical), lower moisture absorption, inertness to the environment, and very high durability [8,9,10,11]. Basalt fibers are extracted from this rock through melt extrusion. The rock is first broken into very small pieces, melted, and then made to flow through bushings with many fine orifices to form the fiber. This process does not use any additional materials; the fiber is made with a naturally available material and requires less energy and is cheaper than many other high-performance fibers such as glass, carbon, etc. The chemical composition of basalt fiber is very similar to that of glass fiber [12].

Unlike many other fibers, basalt fiber is known to properly disseminate in the concrete mix when used instead of forming balls and has very high bio-solubility. Its stress–strain behavior is linear up to its failure [13,14,15,16,17]. It is more economic compared with carbon fiber and is more advantageous compared with glass fiber with respect to many criteria (e.g., Young’s modulus, acid resistance, etc.) and has therefore been extensively investigated in many studies in order to explore the possibility of replacing glass fibers in concrete [18,19,20,21,22].

Basalt-fiber-reinforced concrete shows higher splitting tensile strength and toughness as compared to pure concrete, but its compressive strength decreases over time. With increasing fiber length, there is an increase in tensile strength, compressive strength, and toughness, which may be because interfacial bonding increases with increasing surface area [23,24,25,26]. Basalt fiber shows a higher Young’s modulus than glass fiber, and the compressive strength and flexural behavior of basalt-reinforced concrete is found to be superior. However, basalt fibers show lower tensile strength as compared to glass fibers [27]. Basalt fibers or fibrous materials are usually coated with a binding agent in order to make them compatible with the concrete. It has been observed that basalt fibers are ‘wetted out’ by these binders well [28,29,30,31]. A higher number of admixtures must be added in order to increase the slump to the required level. This is due to the fact that the fibers have larger surface areas as compared to the aggregates in the mix, and they bond with a higher amount of cement, thereby increasing the viscosity of the mix [32,33].

Basalt fibers are extracted from volcanic rocks and are therefore a purely natural product. They do not involve artificial chemicals or reagents. They have been referred to as a “green material” in the literature. The environmental impact of using such material instead of synthetic material is very significant [33,34].

Using chopped basalt fibers in concrete mix is not the only way of reinforcing. Basalt-woven structures or rebars are other alternatives. They are lighter than other forms of reinforcement, and using a lower amount of basalt fibers/rebars may generate the same strength while considerably reducing the weight of the slabs [34,35]. They can also be cut into the length required more easily with the normal available cutting tools.

In this study, the mechanical properties of basalt-fiber-reinforced concrete slabs were predicted using the finite element analysis. Simulations were conducted using ANSYS by making CAD models of the samples and the obtained results were analyzed. To study the tensile property of basalt-fiber-reinforced concrete, experiments were carried out by making samples of pure concrete and 1%, 2%, and 3% basalt-fiber-(by weight) reinforced concrete. The simulation results were compared with experimental samples as well as with findings of previous studies. The properties were also compared to those of fine and coarse aggregates used in concrete. The influence of basalt fiber as a replacement for stone/aggregates in concrete slabs was investigated.

## 2. Materials and Methods

### 2.1. Materials

Details of the basalt fibers and concrete used are given in Table 1. The basalt fiber was received from the company Basaltex (Wevelgem, Belgium). The standard commercial concrete was purchased from a local market.

The chemical composition of the basalt fiber is given in Table 2.

A qualitative elemental analysis of the basalt fiber determined that SiO_2_ and Al_2_O were the dominant compounds. The content of FeO and Fe_2_O_3_ played a very important role in determining many physico-mechanical properties of basalt fibers, such as density, color (from brown to dull green, depending on the FeO content), lower heat conduction, and better temperature stability compared with those of E-glass fibers. The basalt fibers were pretreated against possible alkali reactions [33,35].

### 2.2. Methods

#### Experimental Samples

**Figure 1:**
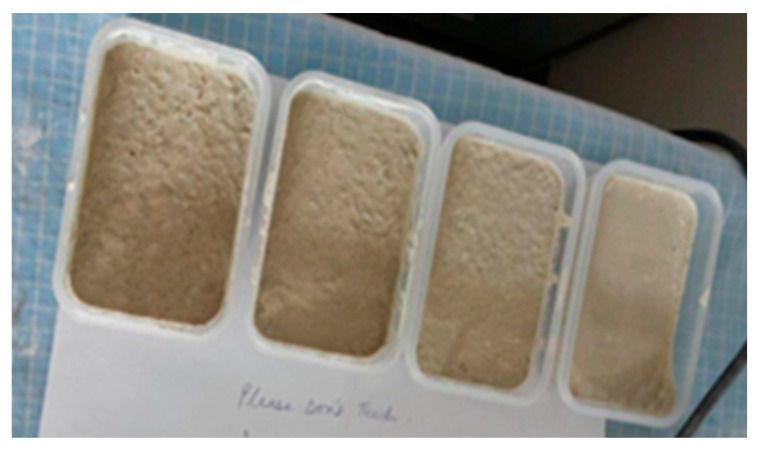
Samples prepared for tensile testing.

Four types of experimental samples were prepared as shown in Figure 1. They were the plain concrete sample, 1% (by weight of concrete) basalt-fiber-reinforced sample, 2% basalt-fiber-reinforced sample, and 3% basalt-fiber-reinforced sample. The concrete samples were prepared by mixing the cement-aggregate mixture with 30% water (by weight of concrete) and different percentages of chopped, short staple basalt fibers (by weight of concrete). The samples had a dimension of 11 cm × 7 cm × 1 cm. These samples were tested under a tensile load.

**Figure 2:**
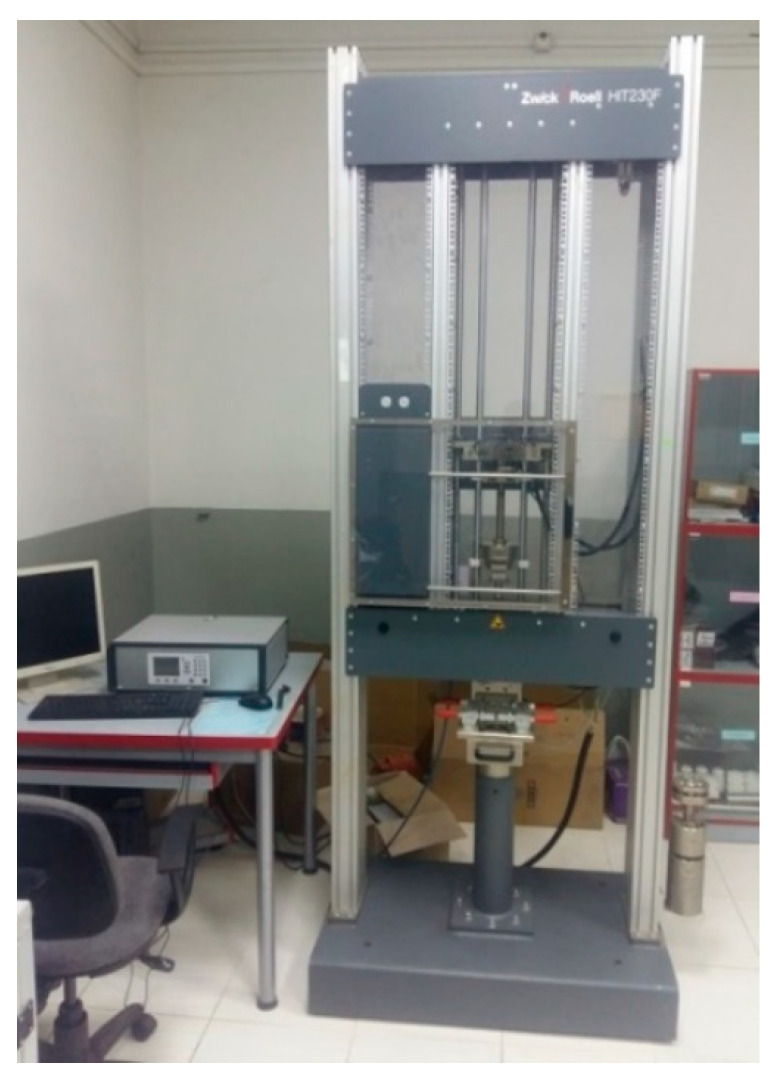
Zwick/Roell universal tensile tester.

The tensile testing was conducted on a Zwick/Roell universal tensile tester as per the standard ASTM C1399 [36,37]. Twenty samples sized 11 cm × 7 cm × 1 cm were tested for each experiment. The average of 20 measurements was reported to have significance at a 95% confidence interval. The coefficient of variation was below 5%. The testing device is shown in Figure 2.

The average material property was used for defining the composite/concrete elements. Multi-scale mechanical models were developed in order to predict the properties of the fiber-based composites.

For the theoretical estimation of the mechanical properties of the fiber-reinforced concrete, the corresponding mechanical properties of the fibers and pure concrete were experimentally evaluated. The mechanical properties of the basalt-fiber-reinforced concrete was estimated using a Halpin–Tsai model. This model is commonly used for the prediction of the effective mechanical properties of fiber-reinforced concrete (composites) [36,37,38]. The Halpin–Tsai equation is expressed as:

(1) $$K_{c}=K_{m}\left[\frac{1+\xi \zeta V_{f}}{1-\eta V_{f}}\right]$$

(2) $$\mathrm{With}\;\eta =\left[\frac{\left(K_{f}/K_{m}\right)-1}{\left(K_{f}/K_{m}\right)+\zeta }\right]$$

where,

*K_c_* = the effective mechanical property of the fiber-reinforced concrete;

*K_f_* and *K_m_* are the corresponding fiber and concrete mechanical properties, respectively;

*V_f_* = the fiber volume fraction;

*ζ* = the geometrical parameter, which represents the loading conditions (e.g., uniaxial, biaxial, multiaxial, etc.).

The modeling was carried out using a micro-scale representative volume element (RVE), which was subsequently transferred to a meso-scale RVE of the final composite to calculate the elastic constants by homogenization. Research has been reported about the prediction of mechanical properties in concrete with a given fiber-volume percentage. The predictions were reported to have a very good agreement with the experimental results [39,40].

The FE solution is broken down into the following three stages. This is the basic guideline that can be used for setting up any FEA [39,40,41].

The major steps in preprocessing are given below:

- Defining key points/lines/areas/volumes in the concrete slab.
- Defining element type and material/geometric properties for fiber, gravels, as well as the cementitious matrix.
- Defining mesh lines/areas/volumes as required.

The amount of detail required depends on the dimensionality of the analysis (i.e., 1D, 2D, axisymmetric, and 3D).

Simulations were conducted on the following models to test their tensile strength:

- Plain concrete sample;
- Concrete sample reinforced with 1% basalt fiber;
- Concrete sample reinforced with 2% basalt fiber;
- Concrete sample reinforced with 3% basalt fiber.

In addition to these, some other simulations were also conducted in order to compare the fiber-reinforced concrete with the standard concrete reinforced with aggregates/stones. For such a simulation, the volume fraction of aggregates was considered as 3%. The density of such aggregates was taken from the literature [31,34,38]. The properties of concrete and the aggregates were inputted into the model. All other conditions of loading were the same for the 3% basalt-fiber-reinforced concrete. The parameters were selected as follows:

- Three percent very fine aggregates (average 4 mm) with a density of 2615 kg/m^3^ and a volume equivalent to that of the chopped fibers;
- Three percent coarse aggregates (average 7 mm) with a density of 2660 kg/m^3^ and a volume equivalent to that of the chopped fibers.

**Figure 3:**
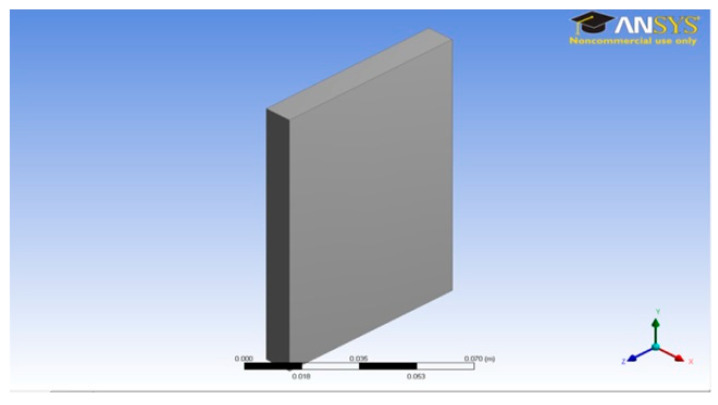
Plain concrete slab model (11 × 7 × 1 cm).

**Figure 4:**
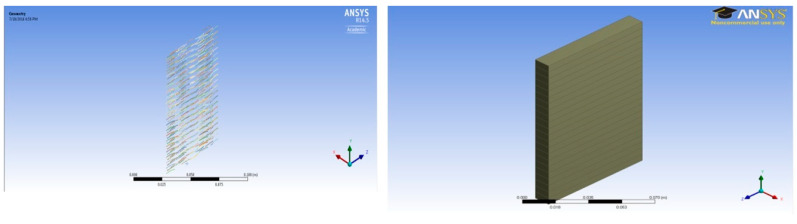
One percent fiber sample model (11 × 7 × 1 cm) and concrete slab.

**Figure 5:**
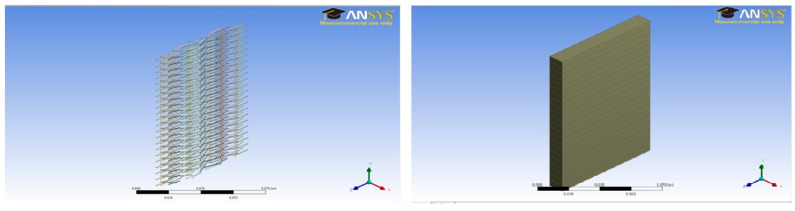
Two percent fiber sample model (11 × 7 × 1 cm) and concrete slab.

**Figure 6:**
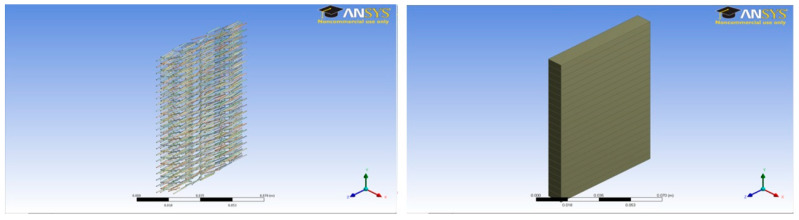
Three percent fiber sample model (11 × 7 × 1 cm) and concrete slab.

**Figure 7:**
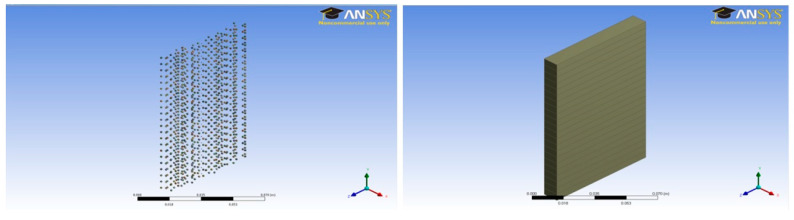
Three percent fine aggregate sample model (11 × 7 × 1 cm) and concrete slab.

**Figure 8:**
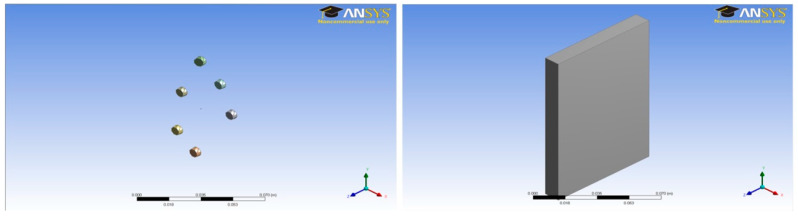
Three percent coarse aggregate sample model (11 × 7 × 1 cm) and concrete slab.

A unit cell was prepared for each case with a dimension of 0.5 × 7 × 1 cm. A total of twenty-two (22) unit cells were stacked one above the other to make the complete concrete slab structures shown in Figure 3, Figure 4, Figure 5, Figure 6, Figure 7 and Figure 8.

The static structural module in ANSYS was used to carry out the simulation in order to compare the tensile strengths of each of the samples. The following simulations were carried out:

- The simulation of all samples with one end fixed and a force of 50 × 10^5^ N acting on the opposite face.
- The simulation of the pure concrete sample with one end fixed and a force of 150 × 10^5^ N acting on the opposite face (the force at which pure concrete the block reached failure in the actual conducted experiment).
- The simulation of the 1% fiber-reinforced concrete sample with one end fixed and a force of 300 × 10^5^ N acting on the opposite face (the force at which the 1% fiber-reinforced concrete sample failed in the conducted experiment).
- The simulation of the 2% fiber sample with one end fixed and a force of 500 × 10^5^ N acting on the opposite face (the force at which the 2% fiber-reinforced concrete sample failed in the conducted experiment).
- The simulation of all the 3% filler-based concrete samples (fibers, fine aggregates, and coarse aggregates) with one end fixed and a force of 700 × 10^5^ N acting on the opposite face (the force at which the 3% fiber sample failed in the experiment conducted).

To determine the advantages of the linear behavior of basalt fiber until failure, simulations were also conducted assuming a yield point before the ultimate failure. The simulations for these are not shown in this paper, but the effects were insignificant.

The input parameters given are as follows:

Young’s modulus of basalt fiber = 89 GPa; Poisson’s ratio of basalt fiber = 0.3.

Young’s modulus of concrete = 17 GPa; Poisson’s ratio of concrete = 0.33.

The tensile yield and ultimate strength values, as well as the compression yield and ultimate strengths were also given as inputs, but they were found to have no effect on the results as explained later in this section.

The basalt fibers, fine aggregates, and coarse aggregates were all given with the same dimensions for comparison purposes. The model was built with a coarse mesh and the simulation was carried out. The distribution of stress along the samples were found for the following parameters in each sample:

Directional deformation (m)—(along the *y*-axis, along which the force was applied).

Strain energy (*U*) is given by the following expression:

*U =* 0.5 × *(V*/*E)* × *S*^2^ (3)

where *U* = strain energy, *V* = volume, *E* = Young’s modulus, and *S* = stress.

This gave a measurement of Young’s modulus of different samples under similar stress conditions. The higher the stored strain energy, the lower the Young’s modulus of the structure (or part of the structure being analyzed).

## 3. Results and Discussion

### 3.1. Experimental Results

**Figure 9:**
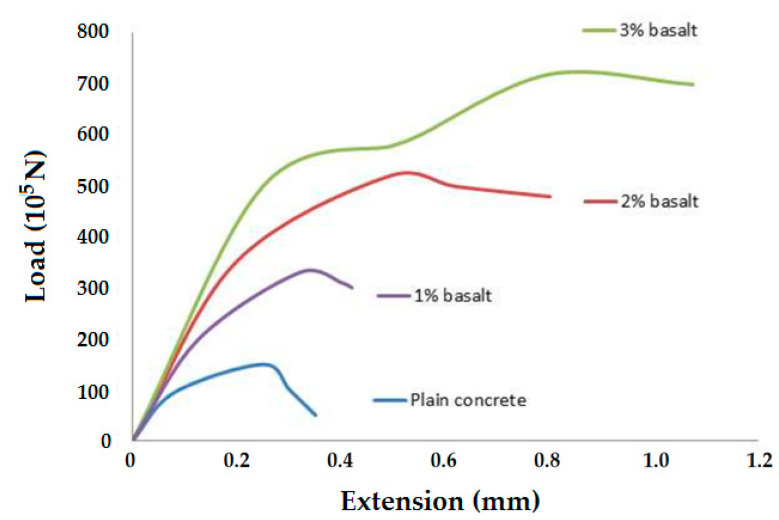
Load-extension curves from tensile testing of experimental concrete slabs.

The experimental samples were tested on a universal testing machine. The average result of 20 measurements was taken. The load extension graph obtained for the samples is given in Figure 9.

**Figure 10:**
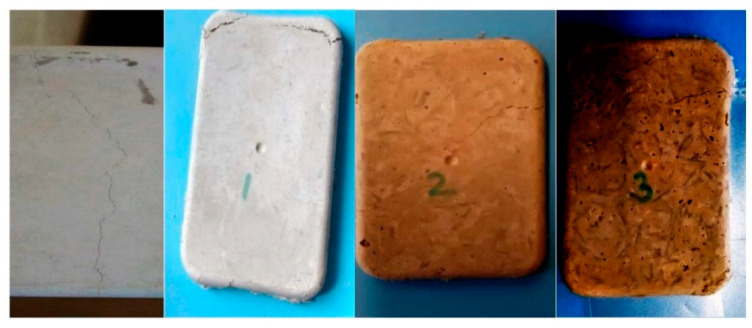
Crack patterns in 0%, 1%, 2%, and 3% basalt-fiber-reinforced concrete samples after tensile testing (pictures formatted so that crack patterns are clearer).

The tensile strength significantly increased as the percentage of the fiber increased. The strength increased by around four times when the fiber percentage was 2%. The elastic modulus also increased when the fiber percentage increased from 0 to 1% but after that, remained almost constant when further increased. The crack patterns were also very different for the sample without the fibers and the samples reinforced with the fibers. The failure/crack path in the case of the samples reinforced with basalt fibers was much more tortuous due to the presence of fibers, not a straight horizontal line (i.e., the shortest path), unlike the case of the pure concrete sample. The overall extension increased for the fiber-reinforced concrete, and it was also proportional to the fiber weight percentage. The samples are shown in Figure 10. The cracking pattern showed that with an increasing weight percentage of basalt fiber, cracking was more difficult. This indicated that basalt fibers are able to more efficiently absorb the tensile stress in concrete.

The peak load and extension are also given in Table 3.

It can be observed that an addition of basalt fiber of up to 3% increased the peak stress in the concrete. Further, the deformation at peak stress was reduced, thus increasing the load-bearing capacity. The basalt-fiber-reinforced concrete showed improved performance as compared to pure concrete as well as fine-/coarse-aggregate-based samples. Similar studies have been reported in the literature where the performance of concrete was improved by an addition of up to 3% microfibers and waste mineral admixtures [42].

The inclusion of micro-silica in small doses has been reported to be superior with respect to the tensile performance of concrete. It has also been reported that micro-fillers enhance the bond strength, which was responsible for the improvement of mechanical performance in concrete composites [43].

### 3.2. Results of Simulations Using FEM

The simulations of concrete samples were carried out based on the defined conditions. The results are shown in Figure 11, Figure 12, Figure 13, Figure 14, Figure 15, Figure 16, Figure 17, Figure 18, Figure 19, Figure 20, Figure 21 and Figure 22.

The model for the plain concrete sample predicted a maximum stress of 6.9 × 10^4^ Pa and a maximum strain 4.2 × 10^−6^ m/m at 50 × 10^5^ N force. The corresponding deformation was 1.36 × 10^−6^ m. These values were quite close to the experimental result. 

The predicted value for maximum stress at 150 × 10^5^ N force was 2.1 × 10^5^ Pa. The maximum strain was predicted as 1.25 × 10^−5^ m/m.

The model for the 1% basalt-fiber-reinforced concrete predicted an increased maximum stress at 50 × 10^5^ N force. The value was predicted to be 4.35 × 10^5^ Pa. The strain was predicted as 4.75 × 10^−6^ m/m.

Simulations were performed for 1% basalt-fiber-reinforced concrete at a load of 300 × 10^5^ N. they were based on the peak load obtained during the experimental trials. The predicted value of maximum stress was 4.35 × 10^5^ Pa. The corresponding strain was predicted as 2.9 × 10^−5^ m/m.

Further simulations were performed for the 2% basalt-fiber-reinforced concrete samples at 50 × 10^5^ N force. The predicted maximum stress was 7.29 × 10^4^ Pa. The maximum strain was predicted as 4.76 × 10^−6^ m/m. The performance was predicted as superior to that of the 1% basalt-fiber-reinforced concrete sample. This also corresponded to the findings of the experimental analysis.

Based on the peak experimental load, the simulations for the 2% basalt-fiber-based concrete were performed at 500 × 105 N force. The maximum stress was predicted as 7.22 × 10^5^ Pa. This was significantly higher than the maximum stress for the 1% fiber-loaded concrete. Further, the stress was higher at a higher load. The maximum strain was predicted as 4.76 × 10^−5^ m/m. It was observed to be slightly higher at higher loading. The performance was similar to that of the results obtained from the experimental samples.

Simulations for the 3% fiber-reinforced sample were carried out at 50 × 10^5^ N force. The maximum stress was predicted as 7.38 × 10^4^ Pa. This was much higher than the stress level for the 1% and 2% fiber-loaded samples. These findings resembled the experimental tests.

Based on the peak experimental load, further simulations were performed for the 3% basalt-fiber-reinforced concrete slabs at a load of 700 × 10^5^ N. The maximum stress was predicted as 10.18 × 10^5^ Pa. This value was significantly higher than the maximum stress for the 1% and 2% basalt-reinforced concrete. The corresponding strain was predicted as 6.66 × 10^−5^ m/m.

For a comparison of the fiber-reinforced concrete with respect to the standard concrete with fine aggregates, simulations were performed at 50 × 10^5^ N force. The maximum stress was predicted as 7.05 × 10^4^ Pa. This stress value was higher than that of the plain concrete but lower than that of even the 1% basalt-fiber-based concrete. Further, the strain was predicted as 4.42 × 10^−6^ m/m, which was lower than that of the basalt-fiber-based samples but slightly higher than that of the plain concrete sample.

The fine-aggregate-based sample was also loaded at 700 × 10^5^ N force in order to compare its performance with the corresponding sample of the 3% basalt-fiber-reinforced concrete slab. It was predicted that the maximum stress would be 7.12 × 10^5^ Pa. This was higher than the maximum stress for the 1% basalt-fiber-based sample. However, it was significantly lower than that of the 3% basalt-fiber-reinforced sample and slightly lower than that of the 2% fiber-based sample. This was an indication that a 2–3% reinforcement of basalt fiber in the concrete would be optimum. It was also established through the experimental analysis that the 3% basalt-fiber-based samples showed a higher stress level than that of the 3% fine-aggregate-based concrete [44,45]. A higher fiber content resulted in several practical and workability problems. The corresponding strain was predicted as 4.26 × 10^−5^ m/m. This was lower than the predicted stress for the basalt-fiber-reinforced concrete. This was the result of fiber extensibility, which was higher than that of the stones/aggregates.

Simulations were performed on concrete samples reinforced with coarse aggregates. The loading condition was set as 50 × 10^5^ N. The maximum stress was predicted as 6.96 × 10^4^ Pa, which was lower than that of all the samples reinforced with basalt fiber. The maximum strain was predicted as 4.31 × 10^−6^ m/m. Thus, coarse aggregates could be successfully replaced with basalt fibers comprising up to 3% of the overall volume in a concrete mix.

The sample with 3% coarse aggregates was also simulated under 700 × 10^5^ N force. The maximum stress of 6.58 × 10^5^ Pa was predicted. It was significantly lower than the maximum stress in samples reinforced with 2% and 3% basalt fibers. Although, it was higher than that of the pure concrete sample. This indicated that coarse aggregates can be useful for the reinforcement of concrete; however, much better performances can be achieved by replacing these with basalt fibers comprising up to 3% of the overall volume. The maximum strain for the coarse-aggregate-based sample was predicted as 3.88 × 10^−5^ m/m. This was smaller than that of the basalt-fiber-based samples with 2% and 3% loading. This was an indication of higher deformability under loading when there was 2% and 3% basalt fiber in concrete. Such behavior in basalt-fiber-reinforced concrete can prove beneficial under severe loading conditions, e.g., in the case of earthquakes. Similar results have been reported in the literature that a hybrid effect of fibers and fillers improved the crack resistance in reinforced composites and concretes. This performance was dependent on the dosing percentage of the micro-fillers [44,45].

The results of the simulation at 50 × 10^5^ N tensile force are summarized in Table 4.

The results of the simulation based on the corresponding load values obtained from the experimental tensile tests are summarized in Table 5.

**Figure 23:**
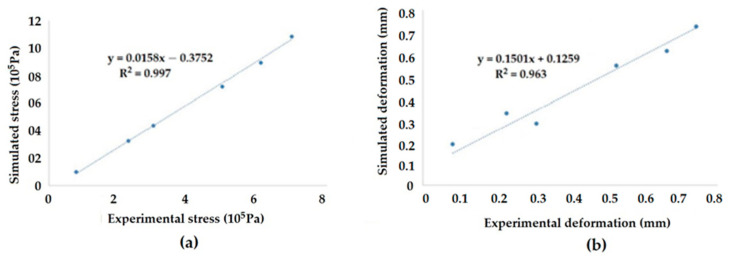
Correlation of results from simulation and experimental samples (**a**) stress and (**b**) deformation.

The meshing used for the FEM was relatively coarse (because of the limitation of the number of nodes for which computation could be performed on the software). Although this did not have much effect on the amount of deformation, the other parameters (strain energy, normal stress, and normal strain) may have varied because of the difference in meshing, and thus the values may have been more accurate and precise. But it can be clearly observed that the trend was similar and independent of the meshing density/intensity. The conditions given in the experiment and the simulations were similar; therefore, the values obtained through the simulation were validated with the obtained experimental values. Thus, it is important to note that the trends were found to be similar to those shown in Figure 23. This indicated that both the computational simulation as well as the experiments were accurately conducted. The validation of the predicted results with the experimental analysis was successful. The error was limited to a maximum of 3%.

It can be observed from the results of the simulation that regarding 50 × 10^5^ N force, the deformations in all the samples were almost the same (as in the case of the experimental samples). The Young’s modulus increased between the 0% and 1% basalt-fiber-based samples but remained almost the same for the 2% and 3% fiber-reinforced samples. The same behavior was also observed from the experiments (the strain energies were compared to compare the Young’s moduli). The force just-before-failure can also be noted (i.e., 150 × 10^5^ N for pure concrete, 300 N for 1% fiber concrete, 500 × 10^5^ N for 2% fiber, and 700 × 10^5^ N for 3% fiber-based concrete). Maximum deformation was obtained for the 3% basalt-fiber-reinforced concrete, followed by the 2% fiber, 1% fiber, and pure concrete slabs. Therefore, it can be concluded that the values significantly varied when the fiber weight percentage was increased from 0% to 1%, but after that, they remained fairly similar, especially for those of the 2% and 3% basalt-fiber-reinforced samples (from 50 × 10^5^ N force).

Other simulations were conducted in order to understand the influence of fillers, e.g., 3% basalt fiber, 3% fine aggregates, or 3% coarse aggregates. The behaviors of basalt-fiber- or fine-aggregate-reinforced concrete slabs were very similar. However, the 3% coarse-aggregate-reinforced sample showed very different results (they were to some extent similar to those of the pure concrete slabs in the 50 × 10^5^ N force category). This seemed to prove that the basalt fiber distribution in the concrete mix influenced the strength, but the fiber structure itself did not affect the mechanical properties. The simulations for the 3% coarse-aggregate-reinforced concrete were conducted using a finer mesh, since the results were not obtained for the same coarse mesh used for other samples, but the values were comparable with those obtained for the pure concrete slabs with fine mesh. There was not much improvement of mechanical performance in the case of the coarse aggregates.

The tensile ultimate and yield points and compression ultimate and yield points of both fiber and cement were also given as input parameters, but it was observed that they did not have much influence on the obtained results.

## 4. Conclusions

The experimental results showed that increasing the percentage of basalt fiber in concrete increased the tensile strength by a significant amount and also improved the crack propagation patterns/tortuosity (it had better elastic behavior). From the simulations, it was also established that the percentage of basalt fiber (or aggregates) did not seem to make much difference when the percentage was increased from 1% to 3% (especially between 2% to 3%), but the increase from 0% to 1% significantly influenced the mechanical behavior. Maximum stress was predicted for 3% basalt-fiber-reinforced concrete samples. The results were found to be similar to those reported in the literature in this area. The simulations were further validated with experimental samples with relatively higher accuracy. The error of prediction was limited to 3%. Moreover, the distribution of the filler material (fiber or aggregates) in the sample influenced the mechanical properties, as observed from the 3% coarse aggregate and pure concrete samples. The fiber’s internal structure itself did not have much effect on the mechanical properties of the concrete sample (shown by similar results for fine aggregates and fibers). The simulation software only considered the values of Young’s modulus and Poisson’s ratio for fiber-reinforced concrete. Changing the input parameters into ultimate tensile and compression strength or yield point strength did not affect the results.

## Figures and Tables

**Figure 11:**
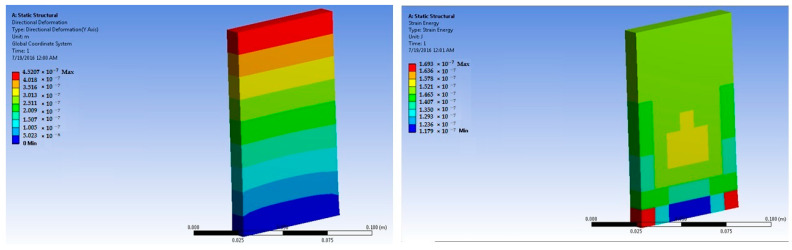
Plain concrete slab at 50 × 10^5^ N force (deformation and strain energy).

**Figure 12:**
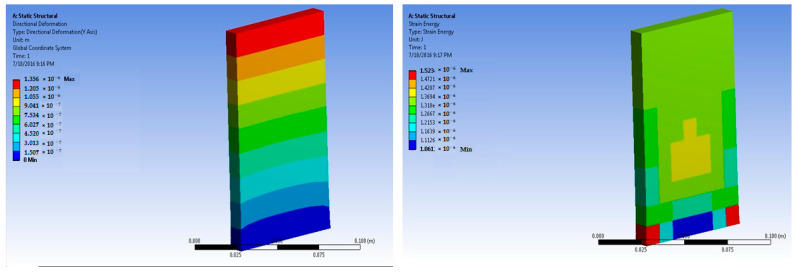
Plain concrete slab at 150 × 10^5^ N force (deformation and strain energy).

**Figure 13:**
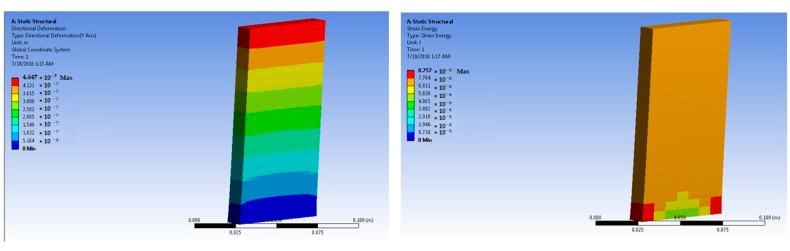
One percent basalt-fiber-reinforced concrete slab at 50 × 10^5^ N force (deformation and strain energy).

**Figure 14:**
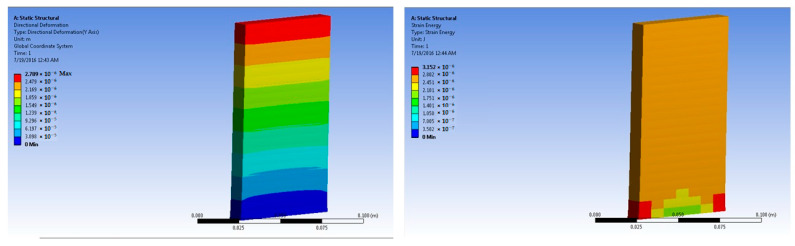
One percent basalt-fiber-reinforced concrete slab at 300 × 10^5^ N force (deformation and strain energy).

**Figure 15:**
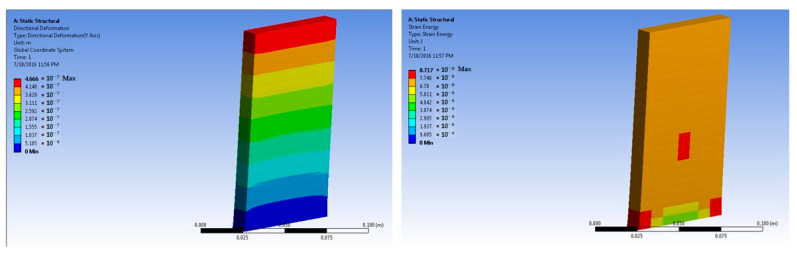
Two percent basalt-fiber-reinforced concrete slab at 50 × 10^5^ N force (deformation and strain energy).

**Figure 16:**
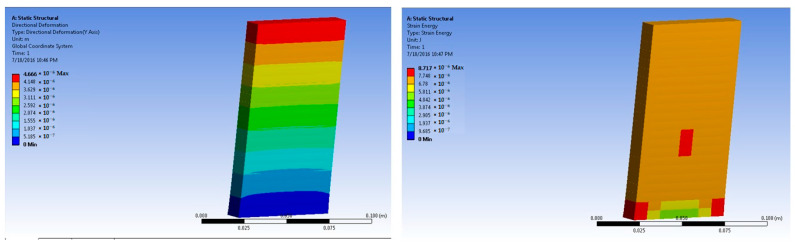
Two percent basalt-fiber-reinforced concrete slab at 500 × 10^5^ N force (deformation and strain energy).

**Figure 17:**
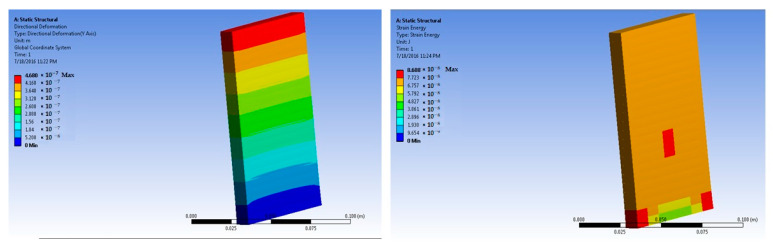
Three percent basalt-fiber-reinforced concrete slab at 50 × 10^5^ N force (deformation and strain energy).

**Figure 18:**
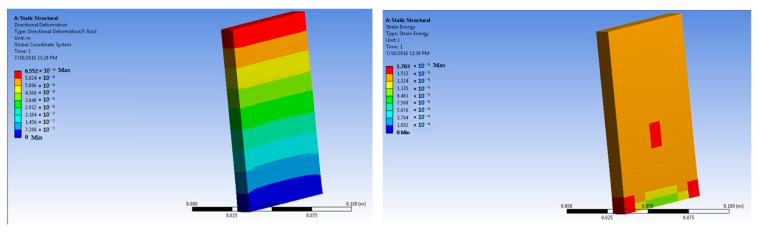
Three percent basalt-fiber-reinforced concrete slab at 700 × 10^5^ N force (deformation and strain energy).

**Figure 19:**
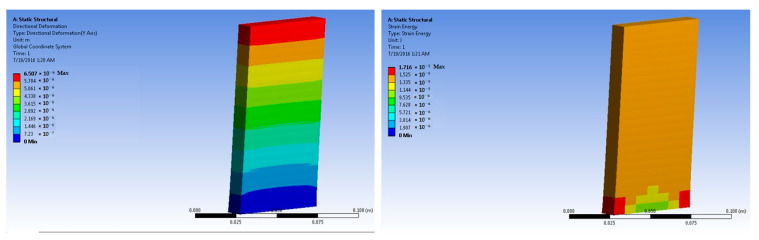
Three percent fine-aggregate-based concrete slab at 50 × 10^5^ N force (deformation and strain energy).

**Figure 20:**
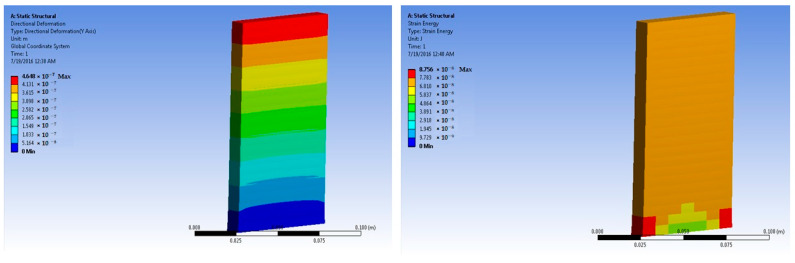
Three percent fine-aggregate-based concrete slab at 700 × 10^5^ N force (deformation and strain energy).

**Figure 21:**
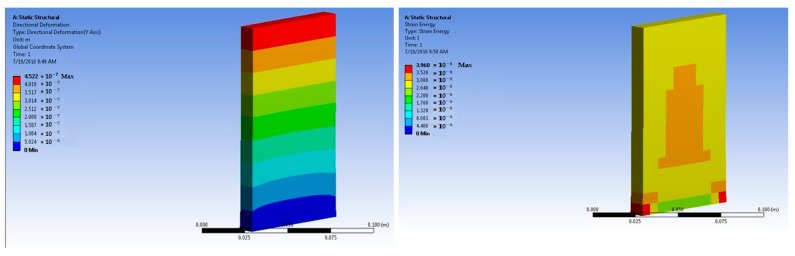
Three percent coarse-aggregate-based concrete slab at 50 × 10^5^ N force (deformation and strain energy).

**Figure 22:**
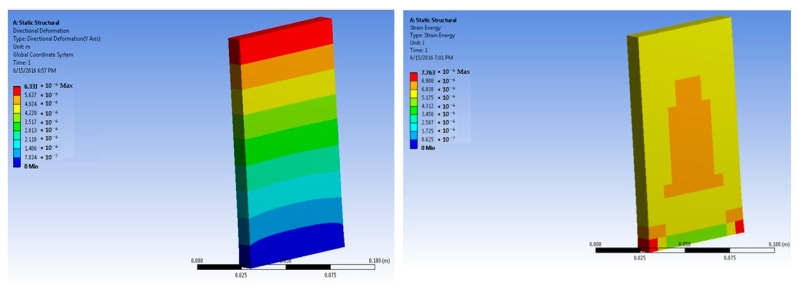
Three percent coarse-aggregate-based concrete slab at 700 × 10^5^ N force (deformation and strain energy).

**Table 1:** Specifications of the basalt fiber and concrete used.

Properties	Basalt Fiber	Concrete
Length	1 cm	
Diameter	0.5 mm	
Grade		M30
Density	2650 kg/m^3^	2240 kg/m^3^
Young’s modulus	89 GPa	17 GPa
Poisson’s ratio	0.3	0.33
Tensile yield strength	4560 MPa	0.142 MPa
Compressive yield strength	49.5 MPa	24.13 MPa
Ultimate tensile strength	4840 MPa	2.2 MPa
Ultimate compressive strength	45.326 MPa	40 MPa

**Table 2:** Chemical composition of the basalt fiber used.

Chemical Composition of Basalt Rocks	%
SiO_2_	52.8
Al_2_O_3_	17.5
Fe_2_O_3_	10.3
MgO	4.63
CaO	8.59
Na_2_O	3.34
K_2_O	1.46
TiO_2_	1.38

**Table 3:** Experimental results for the mechanical behavior of concrete.

Type of Concrete Slab	Experimental Stress (10^5^ Pa)	Deformation at Peak Stress (mm)
Pure concrete slab	0.52	0.75
1% basalt-fiber-reinforced concrete slab	3.64	0.67
2% basalt-fiber-reinforced concrete slab	6.11	0.22
3% basalt-fiber-reinforced concrete slab	7.68	0.13
3% fine-aggregate-reinforced concrete slab	2.65	0.31
3% coarse-aggregate-reinforced concrete slab	5.52	0.51

**Table 4:** Simulation results for mechanical behavior of concrete at 50 × 10^5^ N tensile force.

Type of Concrete Slab	Maximum Deformation (m)	Strain Energy (J)	Normal Stress (Pa)	Normal Strain (m/m)
Pure concrete slab	4.52 × 10^−7^	1.53 × 10^−7^	6.9 × 10^4^	4.2 × 10^−6^
1% basalt-fiber-reinforced concrete slab	4.64 × 10^−7^	7.79 × 10^−8^	7.2 × 10^4^	4.75 × 10^−6^
2% basalt-fiber-reinforced concrete slab	4.67 × 10^−7^	7.75 × 10^−8^	7.29 × 10^4^	4.76 × 10^−6^
3% basalt-fiber-reinforced concrete slab	4.68 × 10^−7^	7.74 × 10^−8^	7.38 × 10^4^	4.76 × 10^−6^
3% fine-aggregate-reinforced concrete slab	4.65 × 10^−7^	8.76 × 10^−8^	7.05 × 10^4^	4.42 × 10^−6^
3% coarse-aggregate-reinforced concrete slab	4.52 × 10^−7^	3.96 × 10^−8^	6.96 × 10^4^	4.31 × 10^−6^

**Table 5:** Simulation results of mechanical behavior of concrete at tensile failure load (load value obtained from the experiment value of peak load just before failure).

Type of Concrete Slab	Maximum Deformation (m)	Strain Energy (J)	Normal Stress (Pa)	Normal Strain (m/m)
Pure concrete slab (150 × 10^5^ N)	1.36 × 10^−6^	1.32× 10^−6^	2.1 × 10^5^	1.25 × 10^−5^
1% basalt-fiber-reinforced concrete slab (300 × 10^5^ N)	2.79 × 10^−6^	2.85 × 10^−6^	4.35 × 10^5^	2.9 × 10^−5^
2% basalt-fiber-reinforced concrete slab (500 × 10^5^ N)	4.67 × 10^−6^	7.8 × 10^−6^	7.22 × 10^5^	4.76 × 10^−5^
3% basalt-fiber-reinforced concrete slab (700 × 10^5^ N)	6.55 × 10^−6^	10.52 × 10^−6^	10.18 × 10^5^	6.66 × 10^−5^
3% fine-aggregate-reinforced concrete slab (700 × 10^5^ N)	6.51 × 10^−6^	10.72 × 10^−6^	7.12 × 10^5^	4.26 × 10^−5^
3% coarse-aggregate-reinforced concrete slab (700 × 10^5^ N)	6.33 × 10^−6^	7.76 × 10^−6^	6.58 × 10^5^	3.88 × 10^−5^

## Data Availability

Not applicable.
